# Item-Level Psychometrics for the Functional Gait Assessment in Persons With Stroke

**DOI:** 10.1016/j.arrct.2025.100452

**Published:** 2025-04-01

**Authors:** Bryant A. Seamon, Steven A. Kautz, Mark G. Bowden, Jesse C. Dean, Chris M. Gregory, Richard R. Neptune, Craig A. Velozo

**Affiliations:** a Ralph H. Johnson VA Health Care System, Charleston, SC; b Division of Physical Therapy, Department of Rehabilitation Sciences, College of Health Professions, Medical University of South Carolina, Charleston, SC; c Department of Health Sciences and Research, College of Health Professions, Medical University of South Carolina, Charleston, SC; d Clinical Integration and Research Division, Brooks Rehabilitation, Jacksonville, FL; e Walker Department of Mechanical Engineering, The University of Texas at Austin, Austin, TX; f Division of Occupational Therapy, Department of Health Professions, College of Health Professions, Medical University of South Carolina, Charleston, SC

**Keywords:** Balance, Gait, Measurement applied, Rehabilitation, Stroke

## Abstract

**Objective:**

To determine the item-level psychometrics of the Functional Gait Assessment (FGA) for persons with chronic stroke and create a keyform (or score sheet) for clinicians.

**Design:**

Retrospective cohort.

**Setting:**

Archival item-level data from a research database.

**Participants:**

One-hundred-one ambulatory persons (N=101) with chronic stroke (44% women, 58% right hemiparesis, average age 59y, lower extremity Fugl-Meyer 25, and overground self-selected walking speed 0.76 m/s).

**Interventions:**

Not applicable.

**Main Outcome Measures:**

A principal component analysis of the residuals from the Andrich Rating Scale Model (RSM) was used to evaluate unidimensionality and item local dependence. The RSM was also used to examine the rating scale structure, item and person fit, item difficulty hierarchy, and person separation index and to generate a keyform.

**Results:**

Principal component analysis of the residuals confirmed the FGA’s unidimensionality and that no items had local dependence. The category rating scale met the criterion and advanced monotonically. The item difficulty hierarchy was similar to that of community-dwelling older adults. The sample’s mean ability level (ie, person measure) was 0.28 logits (SE=0.63). The FGA had high person reliability (0.90) despite 10% of persons misfitting. There were no floor or ceiling effects, and the FGA separated people into 4 strata. The scored FGA keyform visually showed an individual’s response pattern relative to their measure value.

**Conclusion:**

Rasch analysis supports the use of the FGA to measure walking balance ability in ambulatory persons with chronic stroke. An FGA keyform can provide instantaneous interval measurement for individuals.

Walking balance ability is commonly measured for persons with stroke by the Functional Gait Assessment (FGA). The FGA has 10 items that challenge an individual’s performance across a span of daily walking tasks varying in difficulty.^1^ Items are rated on a 4-category rating scale by a clinician observing the individual’s performance for each task. FGA performance criteria are robust to variability, evidenced by having high inter- and intrarater reliability without requiring specific decision-making guidelines, training, or discussion between clinicians.^1^ An overall FGA score is calculated by summing the items’ ratings (the maximum score is 30).

Conventional classical test theory psychometric properties of the FGA have been reported for persons with chronic stroke.^2^^,^^3^ The FGA has excellent test–retest (ICC=0.95), interrater (ICC=0.94), and intrarater (ICC=0.97) reliability and has strong correlations (r>0.7) with measures of mobility (ie, Barthel Index, Rivermead Mobility Index), walking (ie, gait speed, Functional Ambulatory Category), and balance ability (ie, Berg Balance Scale).^2^^,^^3^ Similar psychometrics have also been reported for other patient populations, including community-dwelling older adults,^4^ persons with vestibular disorders,^1^ and Parkinson’s disease.^5^^,^^6^ The similarity in classical test theory psychometrics across clinical diagnoses commonly seen in outpatient neurologic rehabilitation resulted in a clinical practice guideline recommendation for the FGA to be part of a core set of outcome measures.^7^

Despite professional organization support, clinicians cite significant barriers to standardized measurement use. Common barriers include time (eg, lengthy administration time relative to the value of information gained), lack of clinical relevancy (eg, information is too subjective and items are not relevant to the patient), and limited interpretability (eg, does not contribute to the plan of care).^8^^,^^9^ Addressing clinician-reported barriers to measurement by improving the usefulness of measures, especially because individual test scores appear to have little value to the clinical reasoning process, should be the focus of future research efforts.^10^^,^^11^

Rasch analysis is one way to address these barriers because it generates item-level psychometrics and item difficulty hierarchies that can inform clinicians about an individual patient’s ability based on how they perform across items of various challenges. Models, like the Rasch model for dichotomous items and the Andrich Rating Scale Model (RSM) for polytomous items (ie, items with more than 2 categories), use a probabilistic relationship between a person’s ability and item difficulty, with persons having a high probability of successfully completing items that are easier than their ability and a low probability of success with items that are harder than their ability.^12^ A person’s measure is determined when their ability matches item difficulty (when the patient has a 50% probability of passing an item at a particular rating). This offers several benefits for clinicians, including improved measurement efficiency and score interpretability by identifying where a patient’s ability falls along a continuum. Clinicians can take advantage of this improved clinical utility by using a keyform (or score sheet). Keyforms, first introduced by Linacre,^13^ are Rasch-informed score sheets for generating “instantaneous” measurement values on an interval scale of a person’s performance or response to items. In addition, keyforms visually depict a patient’s response pattern in relation to the item difficulty hierarchy, which can facilitate the interpretability of scores for clinicians.^10^^,^^14^, ^15^, ^16^, ^17^

A previous Rasch analysis of the FGA reported item-level psychometrics for community-dwelling older adults.^18^ The FGA was unidimensional, had an ordered rating scale structure, and had a clinically valid item difficulty hierarchy.^18^ However, there is a need to examine the item-level psychometrics in persons with stroke because the performance-based rating scale used in the FGA may result in a different item hierarchy because of lasting gait and balance impairments that are not commonly found in community-dwelling older adults. The purpose of this study was to examine the item-level psychometrics and item difficulty hierarchy of the FGA and to generate a keyform (or score sheet) for clinicians. It was hypothesized that the FGA would be unidimensional and fit the RSM, as evidenced by an appropriate rating scale structure, good item and person fit, and high person separation.

## Methods

### Data source

This study used a retrospective, cross-sectional research design. Item-level FGA data for 101 individuals with chronic stroke (≥6mo poststroke) were provided by a research database. The database is maintained by the National Institutes of Health Center of Biomedical Research Excellence in Stroke Recovery at the Medical University of South Carolina and approved by the local Institutional Review Board. This study is considered nonhuman subjects research under the revised Federal Policy for the Protection of Human Subjects (Revised Common Rule)^19^ because all data provided from the database were deidentified. Informed consent was not applicable. Individuals were excluded if a lower extremity Fugl-Meyer score or overground self-selected walking speed were not available within a 6-month window from their FGA data collection date. Physical therapists and research staff participated in an internal training session to promote consistency in scoring. Participants were allowed 1 trial for each task. Participants completed FGA items without an assistive device to standardize the protocol across research studies in the database. An ankle brace that freely allowed ankle dorsi- and plantarflexion but provided medial-lateral support was permitted for participants with severe ankle instability in the paretic leg to prevent injury. Demographic data were analyzed with SAS^a^ (version 9.4; SAS Institute).

### Rasch analysis

A Rasch analysis of the FGA with the RSM was done with Winsteps^b^ (version 3.93.1; John Lincare/Winsteps.com) in agreement with the Rasch Reporting Guideline for Rehabilitation Research (RULER) guidelines.^20^^,^^21^

#### Sample size justification

The recommended sample size for evaluating item-level psychometrics with the RSM is based on the desired confidence for stable item calibrations or person measures. A sample of 101 participants is sufficient for obtaining stable item and person calibrations because a sample size of at least 61 participants is recommended to obtain calibrations within 1 logit with a 99% CI for polytomous items.^22^

#### Rating scale structure

The rating scale structure was evaluated against Linacre’s 3 essential criteria: (1) a minimum of 10 observations per rating scale category, (2) rating scale category average measures advance monotonically (ie, demonstrate increasing item difficulty with increasing category value), and (3) outfit mean-squares are <2.^23^ Rating scale categories were collapsed or removed if criteria were not met before continuing the analysis.

#### Unidimensionality

Unidimensionality was tested with principal component analysis (PCA) of the residuals from the RSM. The FGA was considered unidimensional if (1) the RSM model explained ≥40% of the variance, (2) contrast eigenvalue ratios were ≤2, and (3) the observed variance of the first contrast was <4%.^24^ Disattenuated correlations were used to test whether additional factors were potentially measuring different latent variables if any of the criteria were not met. Disattenuated correlations between additional contrasts and the RSM dimension were considered to be measuring the same construct, indicating sufficient unidimensionality for measurement when ≥0.82.^24^

#### Local dependence

Item local dependence was tested with Pearson correlations between item standardized residuals from the PCA. Correlations >0.7 or <−0.7 indicated items were locally dependent.^20^^,^^24^

#### Item and person fit

Items or individuals were labeled as misfitting when fit statistics for infit or outfit had mean-square standardized residuals ≥1.4 and standardized *z* scores ≥2.^23^^,^^25^ Misfitting items were removed to test for the effect on the remaining item fit, and person measures were compared between item sets. Misfitting items were retained if removing items caused additional item misfit and/or person measures were not meaningfully different, evidenced by a Pearson correlation >0.9.^26^

#### Person-item match

The item difficulty hierarchy was used to evaluate the FGA’s theoretical construct validity. Our cutoff to assign a floor and/or ceiling effect was that 15% or more individuals scored the worst or best possible outcome.^27^

#### Person separation index

Person separation was used to quantify how well the FGA can differentiate people into statistically distinct strata. The following formula was used to calculate the number of strata^28^:

Strata=[4×(personseparationindex)+1]/3

#### FGA keyform

Winsteps was used to create a keyform for scoring persons. The sample was separated into terciles, and 1 individual was randomly selected from the middle tercile whose responses fit the RSM to demonstrate an example keyform.

## Results

The average age of the sample was 58.6 years (SD, 12.6). There were 44 women (43.6%) and 42 individuals with left hemiparesis (41.6%). The mean lower extremity Fugl-Meyer score was 25.2 (SD, 5.5), and the mean overground walking speed was 0.76 m/s (SD, 0.29). The average raw score for the FGA was 15.7 (SD, 6.1). Descriptive statistics are presented in table 1.

**Table 1:** Participant demographic information.

Total No. of Participants = 101	
Variable	Summary Statistics
Age (y)	58.6 (12.6, 23-85)
Male (n=57)	56.4%
Female (n=44)	43.6%
Right hemiparesis (n=59)	58.4%
Left hemiparesis (n=42)	41.6%
Lower extremity Fugl-Meyer	25.2 (5.5, 14-34)
Overground self-selected walking speed (m/s)	0.76 (0.29, 0.11-1.4)
Functional Gait Assessment	15.7 (6.1, 6-30)

NOTE. Continuous variables are presented as mean (SD, range). Categorical variables are presented as percentages. Overground walking speeds were collected without the use of an assistive device or orthotic unless a medial-lateral support brace allowing free ankle dorsi- and plantarflexion was used to prevent ankle injury.

### Rating scale structure

The FGA’s 4-category rating scale satisfied each of Linacre’s essential criteria.^23^ Each category on the rating scale had more than 10 observations, with outfit mean-square values <2.0, and average category measures advanced monotonically. The rating scale structure is presented in table 2.

**Table 2:** FGA rating scale structure.

Rating Scale Score	Frequency Count (%)	Observed Measure Average	Infit Mean-Square	Outfit Mean-Square
0	131 (13)	−3.53	0.98	0.98
1	341 (34)	−1.17	0.84	0.81
2	370 (37)	1.32	0.95	1.18
3	168 (17)	3.30	1.21	1.19

### Unidimensionality

The FGA met 2 of the 3 criteria for unidimensionality: (1) measures explained 66% of the variance, and (2) the first contrast eigenvalue was 1.78. However, the first contrast explained 5.9% of the variance, which was greater than the 4% threshold. Disattenuated correlations between the first and second factors with the Rasch dimension were *r*=0.90 and *r*=0.86, respectively, indicating that the FGA was sufficiently unidimensional.

### Local dependence

No item pairs in the PCA of the residuals had correlations >0.7 or <−0.7, indicating no local dependence.

### Item and person fit

Three items misfit the RSM model. Removal of the most misfitting item (“Gait with narrow base of support”) did not cause additional misfit, and person measures generated with and without “Gait with narrow base of support” were highly correlated (*r*=0.993); therefore, the item was retained. The FGA had high person reliability (0.90). Ten persons (10%) misfit the RSM, which did not exceed expectations,^29^ and was unlikely to have a concerning effect on item-level psychometrics.^25^^,^^30^ Misfitting persons were retained, and item fit statistics are presented in difficulty order in table 3.

**Table 3:** Item measure order.

Item	Item Number	Measure (Logits)	SE	Infit Mean-Square (*z* score)	Outfit Mean-Square (*z* score)
Gait with narrow base of support*	7	3.17	0.21	1.78 (4.4)	1.52 (2.4)
Gait with eyes closed*	8	2.48	0.20	1.60 (3.8)	1.54 (3.1)
Step over obstacle	6	0.55	0.18	1.10 (0.7)	1.10 (0.8)
Gait level surface	1	0.31	0.18	0.63 (−3.1)	0.64 (−2.9)
Ambulating backwards	9	−0.34	0.19	0.73 (−2.1)	0.74 (−2.0)
Change in gait speed	2	−0.93	0.19	0.83 (−1.3)	0.82 (−1.3)
Gait with horizontal head turns	3	−1.00	0.19	0.83 (−1.3)	0.82 (−1.3)
Steps	10	−1.03	0.19	0.78 (−1.7)	0.92 (−0.5)
Gait with vertical head turns	4	−1.18	0.19	0.75 (−1.9)	0.74 (−2.0)
Gait and pivot turn*	5	−2.04	0.20	0.98 (−0.1)	1.52 (2.7)

NOTE. Items are presented in difficulty order to show their hierarchy, with the hardest item at the top, followed by items in decreasing level of difficulty.

⁎ Indicates misfitting items.

### Person-item match

**Fig 1:**
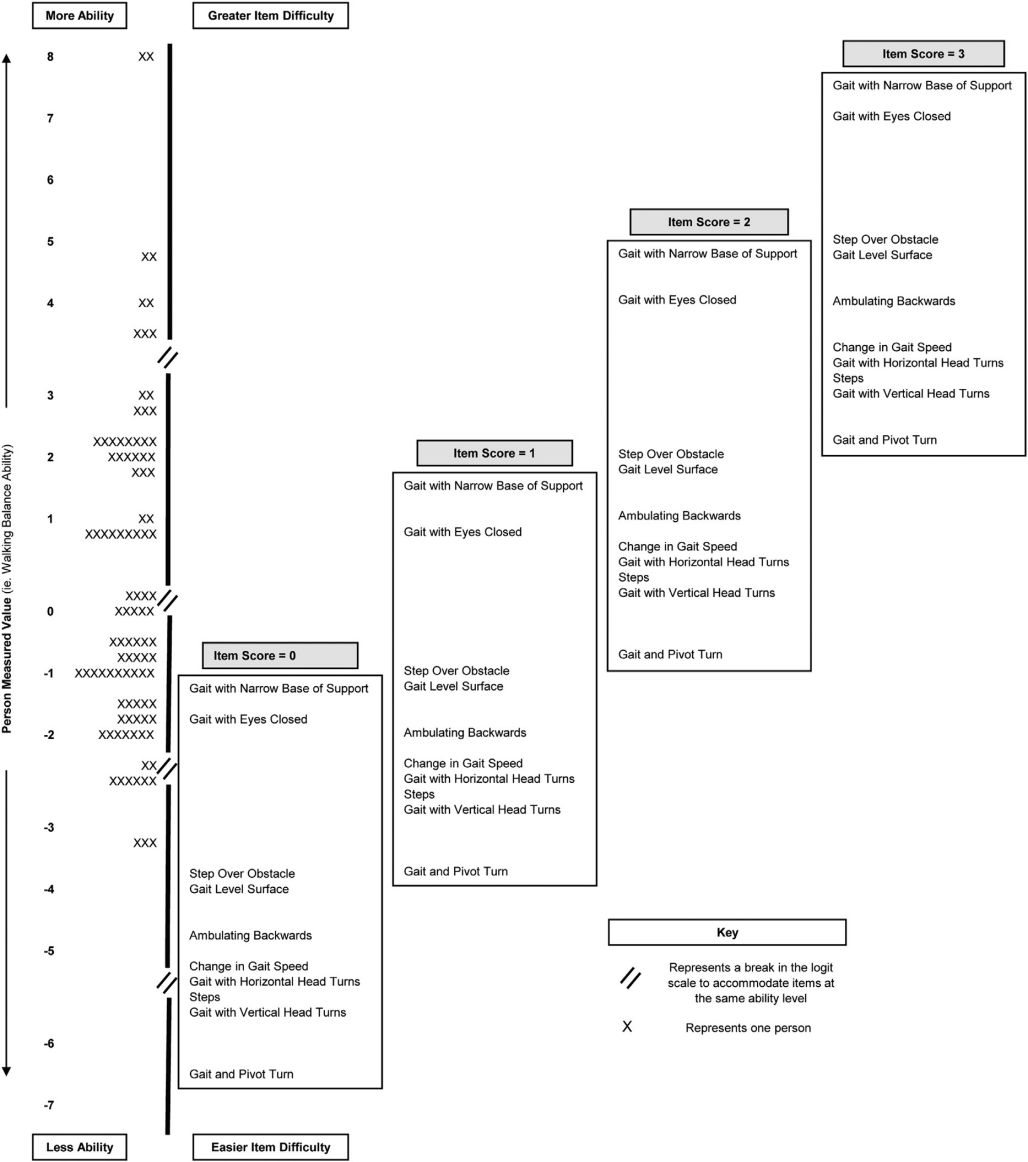
Person-item map. The person-item map presents person ability and item difficulty measures on the same logit scale. The vertical black line represents the logit scale. The distribution of person ability measures (ie, walking balance ability) for the sample is presented to the left of the black line. To the right of the black line, items are presented in a hierarchy from most difficult (top) to least difficult (bottom) at the category rating level. The hierarchy of items is fixed by the RSM. The items are plotted at the corresponding logit measure where the highest probability of an individual receiving a specified value (0, 1, 2, or 3) for that item.

The person-item map is presented in figure 1. The person-item map shows the distribution of people based on ability, also known as person measures (left: low ability; bottom: high ability, top), against item difficulty (right: easy; bottom: hard, top) for each item’s rating scale score (0, 1, 2, and 3). The FGA did not have a ceiling or floor effect. The sample’s mean ability level was 0.28 logits (SE=0.63). This is slightly above the mean difficulty level of the items (anchored at 0 logits), indicating that the item distribution adequately covered the spread of the sample’s walking balance ability.

### Separation index

The FGA differentiated the sample into 4.31 distinct strata with a separation index of 3.01.

### Scoring an FGA keyform

**Fig 2:**
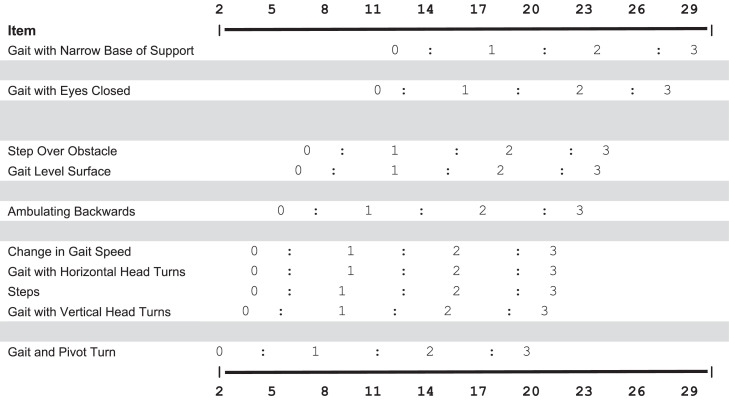
FGA keyform. The top and bottom horizontal axes represent the interval score scale for the FGA. The logit scale produced by the RSM was scaled to match the raw score range for ease of interpretation. The rows of the FGA keyform contain individual items with their category rating values. The items are arranged from easiest to hardest, with the easiest item at the bottom. Items are clustered by similar difficulty, and each cluster is separated by a gray space. The rating scale categories remain the same as the original FGA instructions (ie, 0=severe impairment, 1=moderate impairment, 2=mild impairment, and 3=normal).

The FGA keyform is presented in figure 2. The keyform shows the items in order of difficulty, with the most difficult item listed at the top. Items clustered together and separated by a blank line had overlapping or similar difficulty. The x-axis shows the interval-level measurement scale. The logit scale was scaled to match the original scoring range to enhance clinical interpretation. The row for each item contains the following category rating values: 0=severe impairment, 1=moderate impairment, 2=mild impairment, and 3=normal. Clinicians can circle the rating of each item and draw a vertical line through the “bulk” of the circles to quickly estimate a patient’s measure.^13^ In general, the vertical line should be at the mid-point of the circles’ distribution across the keyform.

**Fig 3:**
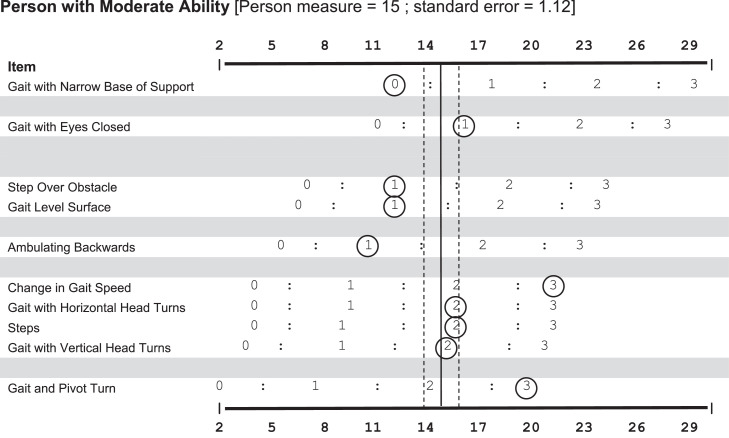
Example of a scored FGA keyform. A scored FGA keyform is presented for a representative individual with moderate walking balance ability. The rating scale from 0-3 matches the original rating scale used with the FGA. The representative individual’s performance on each item is circled. The person’s measure is found by drawing a vertical line through the “bulk” of the circles. The individual’s true ability level (determined by the RSM) is presented by a solid vertical line. The SE associated with the individual’s ability level is presented as 2 dashed vertical lines. The vertical line should move to the right as people improve their balance ability. Observing the response pattern should show clinicians the items that an individual may have difficulty with, given a specific measurement value.

Figure 3 shows a completed FGA keyform for a representative individual with moderate walking balance ability. Actual ratings for each item are circled. The vertical line in figure 3 is reflective of the person’s interval measure from the Rasch analysis. The dashed vertical lines show 2 SEs around this measure and represent the general area where the clinician’s line should be on the keyform.^13^ Visualizing the response pattern to the item difficulty hierarchy provides an additional understanding of the person’s measure relative to their ability, which clinicians could use to guide treatment^10^^,^^31^ and would not be able to be deduced from simply summing the ratings for each item. For example, in figure 3, the individual’s response pattern included a normal (ie, 3) rating for the easiest item, primarily mild impairment (ie, 2) ratings for the next cluster of items, a normal rating (ie, 3) for the next harder item, moderate impairment (ie, 1) for the next 4 items, followed by severe impairment (ie, 0) for the most difficult item, with an overall measure value of 15 (SE=1.12). Ideally, as an individual improves their performance on individual items (ie, an increase in rating category), the vertical line will move to the right of the score sheet, demonstrating an increase in ability.

## Discussion

A PCA of the residuals from the RSM implies that the FGA is sufficiently unidimensional, which supports the hypothesis that the FGA is only measuring a single construct (ie, walking balance ability). A Rasch analysis of the RSM supported the hypothesis that the FGA’s rating scale structure is appropriate and that there was a good match between items and persons’ walking balance ability. Three items potentially misfit the RSM. Fit thresholds have been debated for their accuracy in identifying misfits and can be influenced by the sample.^32^ This study found 3 items marginally exceeded fit thresholds, and the removal of the most misfitting item did not have a significant effect on person measures. Despite potential item misfitting, the item difficulty hierarchy is consistent with clinical and theoretical expectations.^33^

This study’s results were also consistent with a previous Rasch analysis completed on the FGA with data from community-dwelling older adults using the RSM.^18^ Beninato and Ludlow^18^ reported that a PCA of the residuals supported the unidimensionality of the FGA, and the rating scale structure for the FGA items met the recommended criteria for the RSM. They reported similar person reliability (0.83) and did not have a ceiling or floor effect.^18^ Beninato and Ludlow^18^ reported a similar item hierarchy to this study, where “Gait with narrow base of support” was the worst-fitting item. Two differences observed in this study were (1) “Change in gait speed” was a more difficult item for persons with stroke and (2) the item “Gait and pivot turn” was the easiest item for persons with stroke compared with “Change in gait speed,” which was the easiest for older adults.^18^ One possible explanation for these differences is that the persons in this sample did not use an assistive device when completing the FGA. The FGA has 4 items that allow for assistive device use because assistive devices are listed in the rating scale criteria. A second possibility is that persons poststroke have lasting sensorimotor deficits that result in gait deviations, which may not be present in community-dwelling older adults. A third possibility is that the differences are potentially arbitrary because logit estimates are sample-dependent,^34^ and the sample size of both studies was appropriate for estimating item difficulty measures within a 99% CI of 1 logit.^22^ The overall similarity in item difficulty hierarchies between persons poststroke and community-dwelling older adults’ sample abilities suggests that the FGA may be “diagnosis-free,” where measurements could be interpreted similarly across diagnostic groups.

### Implications for research and clinical practice

Findings from this study have important implications for research and clinical practice. First, the findings from this analysis support the validity of the FGA for measuring walking balance in persons with stroke in research and clinical practice. Second, FGA measures for persons with stroke and community-dwelling older adults can be interpreted similarly. This is important for research designs that have older adults as a comparison group. Third, the Rasch analysis provides an interval scale for measurement that can address limitations when using summative scores. Fourth, FGA item-level psychometrics can be used to inform targeted measurement practices like computerized adaptive testing, which can improve efficiency. Lastly, the quantifiable item hierarchy provides a basis for interpreting measures with respect to tasks a person is likely able to do.^31^ Clinicians can use the keyform to visualize this relationship, giving meaning and interpretability to an individual’s measure.^10^^,^^11^^,^^13^^,^^31^ With the keyform, clinicians can quickly see what a patient can or cannot accomplish and which tasks could be plausible treatments (ie, tasks just above the person’s measure or ability). Additionally, keyforms can be used to track patient progress and show patients their expected progression during treatment.^10^^,^^14^, ^15^, ^16^

### Study limitations

There are several limitations to this study. First, the restriction to retrospective data of individuals with chronic stroke associated with 1 research site may limit the generalizability of the results. Second, participants did not use an assistive device when performing FGA items, which may have influenced the item difficulty measures and limited the generalizability of these findings. The similarity of item difficulty measures between Beninato and Ludlow^18^ and this study may indicate that preventing assistive device use did not influence item difficulty measures. Because this remains an empirical question, clinicians should be aware that allowing an assistive device may influence the interpretability of the keyform as it was presented in this study. Third, the measures on the keyform range from 2-29, not 0-30, which may be a barrier to implementation. Rasch measures corresponding to zero and perfect raw scores have infinite SEs.^35^ This may confuse some clinicians who are familiar with 0-30 scores.^36^

## Conclusions

In conclusion, the Rasch analysis supports using the FGA for measuring walking balance ability in persons with chronic stroke. The FGA keyform provides clinicians with a way to use the new interval measurement scale and interpret individual patient measures. This study provides item-level psychometrics for the FGA and additional support for the use of FGA in outpatient neurologic physical therapy settings, as recommended by clinical practice guidelines.^7^

## Suppliers

a. SAS, version 9.4; SAS Institute Inc.
b. Winsteps, version 3.93; John Lincare.

## Disclosure

B.A.S. is currently receiving a subaward from the National Institutes of Health (NIH) Learning Health Systems (LHS) Scholar Program – NIH Learning Health Systems Rehabilitation Research Network (LeaRRn) and a grant from the Department of Veterans Affairs (VISN7 Research Development Award). S.A.K. is currently receiving grants from the NIH (grant nos. P20 GM109040-10, U54 GM104941-10, P2C HD086844-09, and C06 OD036020-01), grants from the Department of Veterans Affairs (grant nos. 1IK6 RX003075, 1IK2 RX003540, 1I01 RX003146-01A1, and I21 RX003881-01A1), an internal grant from the Medical University of South Carolina (award amount $200,000), and an industry grant from Helius Medical Technologies (award amount $164,258). J.C.D. is currently receiving a grant from the NIH (grant no. R01 HD103923), a grant from the Department of Veterans Affairs (grant no. I01 RX004545), and a grant from the National Science Foundation (grant no. 2242812). R.R.N. is currently receiving funding from the Congressionally Directed Medical Research Program (grant no. W81XWH2010164) and the Department of Veterans Affairs (grant no. 1I01 RX003138). C.M.G. was also receiving a grant from the NIH (grant no. R01HD095137) during the time of this project. The other authors have nothing to disclose.
